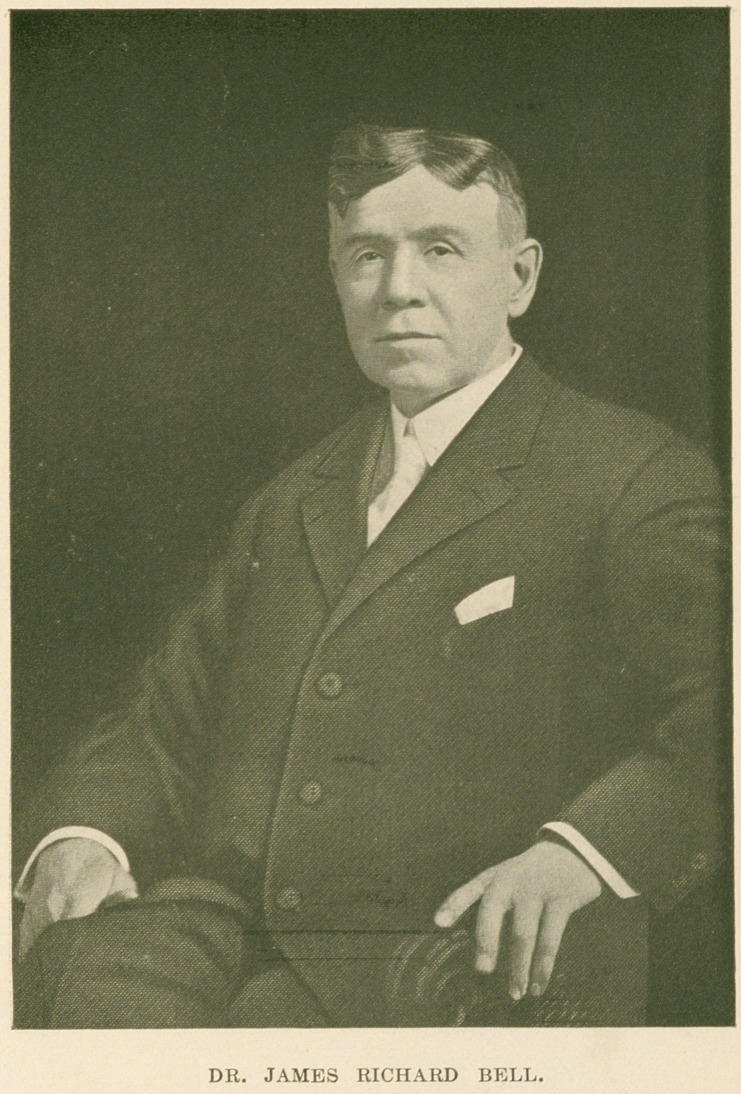# Dr. James Richard Bell

**Published:** 1913-01-15

**Authors:** 


### OBITUARY.

### DR. JAMES RICHARD BELL.

Dr. James Richard Bell, a well-known dentist of Cleveland, Ohio, died November 29th, 1912, after a brief illness due to pneumonia.

DR. JAMES RICHARD BELL.

Dr. Bell's parents were pioneer settlers of Geauga County, but the son was born in Cuyahoga County, February 1st, 1853. He was educated at Geauga Seminary and Hillsdale College, Michigan.

In 1872 he entered the offices of Drs. J. E. and B. F. Robinson of Cleveland, and at the same time took special courses at the Cleveland Medical College. He graduated from the Ohio College of Dental Surgery in Cincinnati, March 1876. He began practice immediately in Cleveland, and remained in continuous practice there thirty-six years until his death.

Dr. Bell was active in dental societies and was always willing to perform any duty that would improve the society or his beloved profession. He wrote a number of articles which were given prominence in dental journals, and was an able clinician at meetings of dental societies.

Dr. Bell was inventive and constructed many things for his personal use. He frequently demonstrated these devices before the dental societies to which he belonged. Among these were a set of trays for taking impressions in wax or dental lac when making gold or porcelain inlays.

These trays were made with 26 gauge tin cut to cover borders of cavity and soldered to No. 12 or 14 gauge wire for handles. They are very useful especially for cervical cavities. A matrix set consisting of a punch, wrench and bolt to be used with thin brass or other suitable metal for the matrix is also very serviceable.

For the surgical removal of gum tissues overlying third molars he devised several special forms of scissors and a tenaculum and lance to facilitate operations in such cases.

Dr. Bell was a fine operator and succeeded in making good and beautiful gold fillings. In handling gold foil he was dextrous and his work was of the substantial sort. We consider Dr. Bell a most thoroughly practical man in all departments of dentistry.

He was so anxious to have the medical profession recognize the importance of dental diseases that he lectured for ten years to the students of the Homoepathic Medical College upon diseases of the teeth and related tissues.

He was one of the charter members of the Cleveland Dental Society and was its President in 1894, and he was President of the Northern Ohio Dental Association in 1886.

He was a modest man, yet he held high ideals tenaciously and could always be found on the right side of all moral questions. During his entire professional life he strove to do the best he could for suffering humanity and succeeded in attracting a large and appreciative practice by his ability and devotion to the needs of his patients. Dr. Bell was interested in and successful in various commercial pursuits. He was an active member of the Chamber of Commerce, he was also a vestryman and trustee of the Reformed Episcopal Church. His immediate family consisted of wife and two children.

The feeling of the members of the dental profession could be well summed up in the expressed Resolutions that was adopted by the Ohio State Dental Society at a recent meeting which are as follows:

*Whereas:* Our Heavenly Father has, in his wisdom, removed from our midst our friend and associate James R. Bell, and

*Whereas:* His long association with our Society and his social intercourse had endeared him to us, and

*Whereas:* We shall miss his presence and helpful influence, be it

*Resolved:* That we, the members of the Ohio State Dental Society, in meeting assembled in Cincinnati, December 4, 1912, extend to his widow and family our heartfelt sympathy in their bereavement and assure them that we, with them, feel his loss and deplore the fact that we, in the future, shall miss his kindly presence and helpful influence in our meetings; and be it further

*Resolved:* That this action be spread upon the minutes of our Society and that the secretary transmit to his family a copy of the same.

(signed)

J. R. OWENS, for the Committee.

The above was adopted by the Society December 4th, 1912, by a standing vote.

F. R. CHAPMAN, *Secretary.*

## Figures and Tables

**Figure f1:**